# Association Study of the Heat Shock Protein 90 Alpha (*HSP90AA1*) Gene Polymorphisms with Schizophrenia in a Polish Population

**DOI:** 10.3390/genes16091092

**Published:** 2025-09-16

**Authors:** Malgorzata Kowalczyk, Aleksander J. Owczarek, Krzysztof Kucia, Maja Hasterok, Renata Suchanek-Raif, Monika Paul-Samojedny, Weronika Lakomy, Jan Kowalski

**Affiliations:** 1 Department of Medical Genetics, Faculty of Pharmaceutical Sciences, Medical University of Silesia, Jedności 8, 41-200 Sosnowiec, Poland; 2 Health Promotion and Obesity Management Unit, Department of Pathophysiology, Faculty of Medical Sciences in Katowice, Medical University of Silesia, Medyków 15, 40-752 Katowice, Poland; 3 Department of Adult Psychiatry, Faculty of Medical Sciences in Katowice, Medical University of Silesia, Ziołowa 45, 40-635 Katowice, Poland

**Keywords:** *HSP90AA1*, association study, polymorphism, schizophrenia, 5-factor PANSS

## Abstract

**Background/Objectives:** Schizophrenia (SCZ) is a highly heritable mental disorder with a complex polygenic genetic architecture. The heat shock protein 90 alpha (HSP90α), encoded by the *HSP90AA1* gene, is a molecular chaperone that is required for the proper folding and activity of many of the client proteins that are involved in numerous essential cellular pathways. In addition to its general chaperone activity, HSP90α plays a role in other neuronal contexts and was found to have an altered expression in SCZ, which makes *HSP90AA1* an attractive gene for association studies. The aim of this study was to determine whether the *HSP90AA1* polymorphisms (rs8005905, rs10873531, rs11621560, rs4947 and rs2298877) are involved in the risk of developing SCZ and its clinical picture in a Polish Caucasian population. **Methods**: A total of 1088 unrelated subjects (409 patients and 679 healthy controls) were included in the study. The SNPs were genotyped using a TaqMan 5′-exonuclease allelic discrimination assay. The results of the Positive and Negative Syndrome Scale (PANSS) were presented in the five-dimensional model. **Results**: None of the SNPs were associated with a predisposition to developing SCZ in either the single-marker or haplotype analysis including the results of gender-stratified analyses. However, the genotypes of rs11621560, rs4947 and rs2298877 SNPs were associated with the emotional distress (EMO) dimension score. **Conclusions**: The results of the present study indicate that *HSP90AA1* variants may have an impact on the psychopathology of SCZ, although larger studies are needed to clarify these findings.

## 1. Introduction

Schizophrenia (SCZ) is a complex, phenotypically heterogeneous and highly heritable psychiatric disorder that affects approximately 1% of the population. The symptoms of SCZ are usually grouped into three categories: positive, negative and cognitive [1,2]. Schizophrenia has proven to be highly polygenic with hundreds of distinct genetic loci involved, much of which is attributable to common risk alleles with small effect sizes [1,3]. Accordingly, large-scale genome-wide association studies (GWAS) have identified numerous low-penetrance loci and risk genes for SCZ that are associated with different aspects of the function and development of the central nervous system (e.g., neuronal differentiation, regulation of synapse structure or activity, neurotransmission) as well as immune-related genes [4,5,6]. It has also been shown that schizophrenia risk genes form a densely interconnected molecular network and may act synergistically to regulate various biological processes throughout the brain [7]. However, a substantial part of the genetic architecture of SCZ remains unexplained [8].

The heat shock protein 90 (HSP90) family constitutes a group of molecular chaperones that play an essential role in proteostasis and also in many cellular processes and regulatory pathways [9]. In humans, there are two cytoplasmatic HSP90 isoforms: the stress-inducible HSP90α (HSP90AA1) and the constitutively expressed HSP90β (HSP90AB1). Combined, both isoforms interact with up to 10% of all proteins and display a substantial functional overlap, although their distinctive functions have also been described [10,11]. HSP90α, encoded by the *HSP90AA1* gene, is widely expressed in the brain and mainly participates in the stress response by preventing the irreversible aggregation and degradation of denatured proteins [12,13,14]. The developing brain is highly sensitive to different stresses that induce HSP90α synthesis, including heat, ischemia, hypoxia and reactive oxygen species [12]. These stressful conditions are among the environmental risk factors for SCZ [15,16]. HSP90 is required for the maturation and proper function of the glucocorticoid receptor (GR), which is involved in the regulation of the hypothalamic-pituitary-adrenal (HPA) axis activity and, as such, is a part of the complex system that controls the response to stress [17,18]. Dysregulation of the HPA axis has been observed in SCZ and was associated with impaired response to psychosocial stressors [19]. Beyond its general chaperone activity, HSP90α plays a role in other neuronal contexts, including neuronal differentiation [12], migration [20], polarisation [21] and neurite outgrowth [22]. HSP90α is not only an intracellular molecular chaperone, but may also be secreted from cells [9,14]. Extracellular HSP90α has been shown to be protective against oxidative stress by inducing an antioxidant response in microglial cells [23].

The HSP90α protein was found to be differentially expressed in the hippocampus (the region strongly implicated in SCZ) of schizophrenic patients compared to healthy controls [24]. HSP90α was also among the differentially expressed proteins that have been identified in the post-mortem dorsolateral prefrontal cortex from low-cumulative-medication SCZ patients. The authors speculated that some of those differentially expressed proteins may represent targets for antipsychotic drugs [25]. In fact, a proteomic analysis found an increased level of HSP90α protein expression in a cell line of human oligodendrocytes after treatment with chlorpromazine [26]. Ishima et al. [22] detected a higher level of HSP90α in PC12 cells after treatment with aripiprazole and speculated that this atypical antipsychotic drug may potentiate an NGF-induced neurite outgrowth via an enhanced HSP90α expression. Moreover, a recent study demonstrated that the antipsychotics olanzapine, aripiprazole and blonanserin recover the decrease of GABAergic interneuron differentiation from NG2(+) cells that is caused by the exposure to the NMDA receptor antagonist MK-801 by increasing the HSP90 level [27]. Taken together, these findings suggest that the enhancement of HSP90α expression may have beneficial effects in patients with SCZ.

It is worth mentioning that *HSP90AA1* is in the SZDB database, which contains the schizophrenia risk genes that have been identified using different methods [28]. Additionally, *HSP90AA1* has been identified as one of 48 SCZ risk genes that have at least 20 direct physical interactions with other SCZ genes [7].

Despite the potential role of *HSP90AA1* in the pathogenesis of SCZ, not a single association study has yet been reported for this gene. Therefore, the aim of the present study was to examine the associations between the five *HSP90AA1* polymorphisms (rs8005905, rs10873531, rs11621560, rs4947 and rs2298877) and the risk of SCZ in a Polish population. We also explored the association between these gene variants and the clinical features of SCZ including the age of onset, the severity of symptoms and suicidal behaviour.

## 2. Materials and Methods

### 2.1. Subjects

A total of 1088 unrelated subjects (n = 409 in the SCZ group: 165 (40.3%) women and 244 (59.7%) men, mean age 42 ± 13 and n = 679 in the control group: 320 (47.1%) women and 359 (52.9%) men, mean age 40 ± 9) were included in the study. All of the participants were Caucasians of a Polish origin living in Upper Silesia. The study protocol was approved by the Bioethics Committee of the Medical University of Silesia (No. KNW/0022/KB1/38/I/12 and KNW/0022/KB1/34/14).

The patients with SCZ were successively recruited starting in 2010 from among inpatients who were being treated at the Department of Psychiatry and Psychotherapy, the Medical University of Silesia in Katowice and the Neuropsychiatric Hospital in Lubliniec; newly diagnosed first-episode psychosis subjects were excluded. All of the patients fulfilled the DSM-IV-TR (*Diagnostic and Statistical Manual of Mental Disorders*, Fourth Edition, Text Revision) criteria for schizophrenia. Exclusion criteria for the patients were as follows: psychotic disorders other than SCZ, mood disorders, anxiety and stress-related disorders, substance-related and addictive disorders as well as a history of neurological, endocrine or autoimmune disorders. The severity of the SCZ symptoms was assessed at the time of hospital admission using the Positive and Negative Syndrome Scale (PANSS) [29]. The five-factor model of the PANSS according to van der Gaag et al. [30] that is composed of positive (POS), negative (NEG), disorganisation (DIS), excitement (EXC) and emotional distress (EMO) dimensions was used. The numerical values of these dimensions were calculated according to the formula provided by the authors using all 30 PANSS items (POS: P1+P3+G9+P6+P5+G1+G12+G16-N5, NEG: N6+N1+N2+N4+G7+N3+G16+G8+G13-P2), DIS: N7+G11+G10+P2+N5+G5+G12+G13+G15+G9, EXC: G14+P4+P7+G8+P5+N3+G4 +G16 and EMO: G2+G6+G3+G4+P6+G1+G15+G16). The mean age of onset of SCZ, defined as the age at which the first psychotic symptoms appeared, was 25.7 ± 6.9. The mean duration of SCZ was 15.9 ± 10.4. This and other detailed clinical information (positive and negative family history of SCZ, suicide attempts) was obtained using in-person interviewing and a review of medical records. All of the patients were assessed to be capable of understanding the study and provided a written consent form prior to enrolment in the study.

The healthy controls were recruited from the volunteer blood donors at the Regional Centre of Blood Donation and Treatment in Katowice. Exclusion criteria for the controls were any neurological disorders, chronic or acute physical illness (infectious, autoimmune or allergic diseases), any current or past mental health problems, a history of substance abuse or dependency and a family history of schizophrenia and other mental disorders (verified during the interview).

### 2.2. SNP Selection Criteria and Genotyping

Five single nucleotide polymorphisms (SNPs) of *HSP90AA1* ((rs8005905 (in exon 2), rs10873531 (in exon 2), rs11621560 (in intron 2), rs4947 (in exon 7) and rs2298877 (in intron 11)) were selected for genotyping. The selection of the SNPs in our study was based on the following criteria: minor allele frequency (MAF) > 0.1 in the European population, assay availability and/or their potential functional significance (rs8005905 is a missense variant). SNP data, including SNP location and allele frequency, were retrieved from a public NCBI database of genetic variation, dbSNP (https://www.ncbi.nlm.nih.gov/snp, accessed on 8 December 2023).

The DNA was extracted from collected blood samples using a Genomic Mini AX Blood kit (A&A Biotechnology, Gdańsk, Poland) according to the manufacturer’s instructions and was quantified spectrophotometrically using a BioPhotometer Plus (Eppendorf AG, Hamburg, Germany).

SNP genotyping was performed using TaqMan SNP Genotyping Assays (Thermo Fisher Scientific, Pleasanton, CA, USA) and either TaqMan Genotyping Master Mix (Thermo Fisher Scientific, Forster City, CA, USA) or TaqPath ProAmp Master Mix (rs4947, Thermo Fisher Scientific, Austin, TX, USA). The assay ID numbers used in this study were: C___2133877_10 (rs11621560), C___3242076_20 (rs2298877), C___2133868_10 (rs8005905), C___2133869_10 (rs10873531) and C___3242071_10 (rs4947). Real-time PCR was performed according to the manufacturer’s recommended protocol using a CFX96 Touch™ Real-Time PCR Detection System (Bio-Rad, Hercules, CA, USA) in a 96-well format. Each 96-well plate contained 91 samples of an unknown genotype, two blank controls (non-template) and three replicate quality control samples that represented the specific genotypes of each SNP. The results were analysed using the allelic discrimination tool of the CFX Manager Software 3.1 (Bio-Rad).

### 2.3. Statistical Analysis

STATISTICA 13.0 PL (StatSoft, TIBCO Inc., Palo Alto, CA, USA), StataSE 13.0 (StataCorp LP, College Station, TX, USA) and R software [31] were used for the statistical analyses. A two-sided *p*-value less than 0.05 was considered statistically significant. The nominal and ordinal data are expressed as percentages, while the descriptive variables are expressed as the mean value ± standard deviation. The two groups were compared using either the Student *t*-test for independent data or the Mann–Whitney U test, depending on the data distribution. The χ^2^ test or Fisher’s exact test were used to evaluate the differences in the genotype distribution and allele frequencies of the *HSP90AA1* polymorphisms between the SCZ patients and controls. The SNPHWE exact test was performed to test the Hardy–Weinberg equilibrium (HWE) of the SNPs in the target population. Odds ratios (ORs) and 95% confidence intervals (CIs) were calculated using a logistic regression analysis. SNPStats (v. 3.21), haplo.stats (v.1.9.7) and SNPAssoc (v. 2.1-2) packages in R (v. 4.5.1)were used to determine the association between the genotypes and SCZ risk in the different genetic models, to estimate the pair-wise linkage disequilibrium (LD), the haplotype frequencies and the association between the genotypes and clinical variables in the different genetic models. The results for the clinical models are presented as the mean with standard error (SE). The association between the genotypes, sex and clinical parameters (PANSS dimensions, age of onset, duration of the disease) was calculated using a two-way ANOVA with Tukey’s post hoc test.

## 3. Results

The initial findings of the study showed that the SCZ patients and the controls were not significantly different in age (42 ± 13 vs. 40 ± 9, *p* = 0.09). There was no difference in age between the genders in the control group (41 ± 9 (males) vs. 40 ± 8 (females), *p* = 0.61), but there was a significant difference in age between the genders (39 ± 12 (males) vs. 46 ± 12 (females), *p* < 0.001) in the study group.

### 3.1. Case-Control Study

*The Genotype and Allele Distributions*. There was no deviation in the genotype frequencies of the five SNPs from the HWE in either the SCZ group (rs11621560 (*p* = 0.46), rs2298877 (*p* = 0.75), rs8005905 (*p* = 0.76), rs10873531 (*p* = 0.80) and rs4947 (*p* = 0.75)) or the control group (rs11621560 (*p* = 0.33), rs2298877 (*p* = 0.79), rs8005905 (*p* = 0.50), rs10873531 (*p* = 0.44) and rs4947 (*p* = 1.00)). There were no statistically significant differences in the distribution of genotypes and allele frequencies between the SCZ and controls for the five *HSP90AA1* polymorphisms that were studied in either the full sample or after stratification according to gender (Table 1). We further explored the potential associations between SCZ and individual polymorphisms using different genetic models (co-dominant, dominant, recessive and over-dominant). Our study showed that none of the SNPs were significantly associated with SCZ in any of the models. However, for the rs2298877 and rs4947, there was a trend toward statistical significance in the recessive model (rs2298877: T/T vs. C/C-C/T, OR 2.37, 95% CI = 0.91–6.21, *p* = 0.07; rs4947: G/G vs. A/A-A/G, OR 2.37, 95% CI = 0.91–6.21, *p* = 0.07) in the males after the gender-stratified analysis.

*Linkage Disequilibrium and Haplotype Analysis*. The D′ values for all of the pairs of SNPs were calculated and linkage disequilibrium (LD) was observed between each pair of markers (all *p* < 0.001): rs11621560 and rs2298877 (D′ = 0.97, r^2^ = 0.34), rs11621560 and rs8005905 (D′ = 0.96, r^2^ = 0.15), rs11621560 and rs10873531 (D′ = 0.96, r^2^ = 0.19), rs11621560 and rs4947 (D′ = 0.97, r^2^ = 0.34), rs2298877 and rs8005905 (D′ = 0.99, r^2^ = 0.44), rs2298877 and rs10873531 (D′ = 0.77, r^2^ = 0.34), rs2298877 and rs4947 (D′ = 1, r^2^ = 0.99), rs8005905 and rs10873531 (D′ = 0.99, r^2^ = 0.78), rs8005905 and rs4947 (D′ = 0.98, r^2^ = 0.44), rs10873531 and rs4947 (D′ = 0.77, r^2^ = 0.34). Among the constructed haplotypes, five had a frequency greater than 1%. The haplotype distribution did not significantly differ between the patients and controls in either the full sample or after the gender-stratified analysis (Table 2). However, for the A–A–C–G–T haplotype, there was a tendency toward statistical significance in the entire sample (*p* = 0.07) and in the males (*p* = 0.07).

### 3.2. Clinical Correlations in Cases

*HSP90AA1 polymorphisms and the clinical presentation of SCZ*. We performed the ANOVA test to examine the possible effect of sex and genotype on the age of onset of schizophrenia, duration of the disease and the individual dimensions of the PANSS scale (five-factor model). We found a significant effect of the rs2298877 and rs4947 genotypes on the EMO factor score (*p* < 0.05). However, the post hoc test showed no significant differences between the particular genotypes. A tendency toward statistical significance was also observed between the genotypes of rs11621560 and the score on the EMO factor (*p* = 0.06).

We further explored the association between the *HSP90AA1* SNPs and the severity of the SCZ symptoms as measured by the PANSS in different genetic models (co-dominant, dominant, recessive and over-dominant). We found significant differences for three SNPs (rs11621560, rs4947 and rs2298877) in the EMO dimension of the PANSS scale (Table 3). The rs11621560 genotypes were associated with the EMO factor score in the recessive model in the entire sample (*p* < 0.05) and the co-dominant (*p* < 0.05), dominant (*p* < 0.05) and recessive (*p* < 0.05) models in females after the gender-stratified analysis. The patients who carried the C/C genotype had higher scores of the EMO factor than those with the A/C and A/A genotypes. The genotypes of rs2298877 and rs4947 were associated with the EMO factor score based on the results of the co-dominant (*p* < 0.05), dominant (*p* < 0.01) and over-dominant (*p* < 0.05) models in the entire sample. We observed that the patients who carried the C/T and T/T genotypes (rs2298877) and the A/G and G/G genotypes (rs4947) had higher mean scores of the EMO factor than those with the most common C/C and A/A genotypes, respectively. We also observed an association between the positive symptom (POS) factor score and the rs2298877 and rs4947 genotypes in the over-dominant model (C/T vs. C/C-T/T and A/G vs. A/A-G/G, *p* < 0.05) in the entire sample (the mean POS scores were C/T and A/G = 24.16 (SE = 0.45), C/C-T/T and A/A-G/G = 22.89 (SE = 0.33)). A strong tendency toward significance was also observed in the dominant model (*p* = 0.052; the mean POS scores were C/C and A/A = 22.90 (SE = 0.35), C/T-T/T and A/G-G/G = 24.00 (SE = 0.41)).

*HSP90AA1 polymorphisms and family history of SCZ*. We also assessed the association between the *HSP90AA1* gene polymorphisms and SCZ in the subgroups classified by a positive or negative family history of SCZ. There were 99 (24.2%) patients with a positive family history of SCZ in our study group, mean age 41.5 ± 12.1 years. We did not find any statistically significant differences in the distribution of the genotypes for any of the SNPs in the total sample or after the gender-stratified analysis between the patients with and without a family history of SCZ.

*HSP90AA1 polymorphisms and suicide attempts.* We also investigated whether *HSP90AA1* polymorphisms might play a role in the susceptibility to suicidal behaviour in SCZ. Among the 409 patients, 76 (18.6%) had attempted suicide, mean age 38.0 ± 10.9. We did not find any statistically significant differences in the distribution of the genotypes for any of the SNPs between the patients with suicide attempts and the patients without attempts in either the total sample or after the gender-stratified analysis. However, we did find that the patients who had attempted suicide had higher mean scores in the EMO factor (23.3 ± 4.8 vs. 21.9 ± 5.0, *p* < 0.05) and lower mean scores in the DIS factor of the PANSS (30.1 ± 7.2 vs. 32.1 ± 6.8, *p* < 0.05) compared to patients who had not attempted suicide.

## 4. Discussion

In this study, we investigated the association between the polymorphisms of the *HSP90AA1* gene and the risk of developing schizophrenia and the clinical features of the disease in a Polish Caucasian population. To the best of our knowledge, this study is the first to evaluate the associations between the *HSP90AA1* polymorphisms and SCZ, although we failed to find any associations based on a case-control study. Interestingly, we found that three SNPs (rs11621560, rs4947 and rs2298877) had an impact on the emotional distress dimension of the 5-factor PANSS.

HSP90 is a dimeric molecular chaperone that is required for the folding and activation of a wide range of the client proteins that are involved in numerous essential cellular pathways [32]. However, the functions of HSP90 in the central nervous system extend far beyond its general chaperon activities, which makes the HSP90 encoding gene an attractive candidate for association studies in neurologic and psychiatric diseases including schizophrenia. The *HSP90AA1* gene has been identified as one of the SCZ risk genes that encode a densely interconnected protein–protein interaction network [7]. The HSP90α protein was also found to have altered expression levels in the hippocampus of schizophrenics compared to controls [24]. It was also among the differentially expressed proteins that were identified in the post-mortem dorsolateral prefrontal cortex from low-cumulative-medication SCZ patients [25].

In the first part of our study (case-control study), we analysed the potential implication of the *HSP90AA1* polymorphisms in the susceptibility to SCZ. Our data did not detect any significant differences in the allele and genotype distribution for any of the SNPs between the patients and controls in either the entire sample or after stratification according to gender. We also did not find any significant associations with SCZ in the haplotype analysis. However, some of the results showed a trend to be statistically significant. Therefore, it is possible that increasing the size of the study population could contribute to the statistical significance, especially in the gender-stratified analysis (although the opposite result is equally possible and the tendency towards statistical significance would then be lost). In the different genetic model analysis, we observed a trend towards statistical significance for two SNPs (rs2298877 and rs4947) in the recessive model in the male sample (homozygous genotypes for the minor alleles were over-represented among the patients). Moreover, the A–A–C–G–T haplotype tended to be significantly associated with SCZ in the entire sample and in the men. As was mentioned earlier, HSP90 regulates the GR activity and abnormalities in GR signalling that lead to a disturbance of the HPA axis homeostasis contribute to psychiatric diseases [19,33]. Numerous studies have demonstrated that there are notable gender differences in the stress response system, including variations in the HPA axis activity and the expression and function of the GR that affect glucocorticoid sensitivity [34,35]. Studies have also revealed both regional and cellular specificity in the GR signalling properties in males vs. females that likely underlie the profound sex differences in stress responsivity [36]. It has also been reported that males had stronger HPA axis responses to a psychological stressor than females [37]. It is possible that the *HS90AA1* gene polymorphisms may contribute to the gender-specific differences in stress susceptibility and SCZ risk. Male-specific genetic associations in SCZ have previously been reported for several genes, including *COMT* [38], *IFNGR2* [39], *TNFR2* [40], *CACNA1C* [41], *VMAT2* [42] and *TRKB* [43].

In the second part of our research, we analysed the individual impact of the *HSP90AA1* polymorphisms (rs8005905, rs10873531, rs11621560, rs4947 and rs2298877) on the clinical parameters of SCZ, such as the age of onset and the severity of symptoms as measured by the PANSS. We used a five-factor model of the PANSS, which is believed to better cluster the individual PANSS items than the original PANSS subscales. Interestingly, the *HSP90AA1* polymorphisms were associated with the severity of the emotional distress (EMO) dimension symptoms of the five-factor PANSS. The ANOVA showed a significant effect of the rs2298877 and rs4947 genotypes on the EMO factor score. Moreover, three SNPs were found to be significantly associated with the EMO dimension score under various genetic models. The rs11621560 genotypes were associated with the EMO factor score in the recessive model in the entire sample. They were also associated with the EMO factor score in the co-dominant, dominant and recessive models in the females after the gender-stratified analysis. The patients who carried the C/C genotype had higher scores of the EMO factor than those with the A/C and A/A genotypes. The genotypes of rs2298877 and rs4947 were associated with the EMO factor score based on the results of the co-dominant, dominant and over-dominant models in the entire sample. We observed that the patients who carried the genotypes rs2298877 C/T and T/T and rs4947 A/G and G/G genotypes had higher mean scores of the EMO factor than those with the most common C/C and A/A genotypes, respectively.

HSP90 is an important glucocorticoid receptor (GR) chaperone that is required for the GR to bind a ligand and become active [44]. It was revealed that both of the human HSP90 isoforms had equal activity in the activation of GR steroid binding [45]. However, HSP90α is stress-inducible and is present at higher levels during stress. The HPA axis activity during the stress response is largely regulated by GR and dysregulation within the GR stress signalling pathway may confer susceptibility to stress in mood and psychotic disorders [46,47]. Of note, the hyperactivity of the HPA axis and increased level of the glucocorticoid hormones are one of the most consistent findings in major depressive disorders and the impaired feedback regulation of the HPA axis is possibly caused by the decreased function of the GR [48,49]. Accordingly, genes related to the proper functioning of GR may be involved in the development of depressive symptoms. For example, polymorphisms of the FKBP51-encoding gene (the co-chaperone of HSP90) have been associated with major depression [50,51,52]. The HSP90 polymorphism was associated with the risk of anxiety among coronary artery disease patients [53]. Three *HSP90AA1* polymorphisms (rs7160651, rs10873531 and rs2298877) were associated with the response of systemic lupus erythematosus patients to glucocorticoids treatment [54], thus indicating that an *HSP90AA1* variation may affect GR function. In this study, we observed significant associations between the *HSP90AA1* rs11621560, rs4947 and rs2298877 polymorphisms and the EMO dimension of the five-factor PANSS. The vast majority of the five-factor models consists of the positive, negative, cognitive/disorganisation, depression/anxiety and hostility/excitement factors [55,56]. In the five-factor model used in our study, the EMO factor captured all five of the original general psychopathology items (G2—anxiety, G6—depression, G3—guilt feelings, G4—tension and G1—somatic concern) that are typically included in the depression/anxiety factor in other five-dimensional models. It is possible that the *HSP90AA1* polymorphisms may be associated with the severity of depression and anxiety symptoms in SCZ. It is worth noting that depressive symptoms are a common feature of SCZ and their presence increases the risk of other symptoms worsening and is a major risk factor for suicide [57,58]. The latter is consistent with our observation because in this study, patients who had attempted suicide had higher mean EMO factor scores compared to patients who had not attempted suicide. Interestingly, popular atypical antipsychotics such as olanzapine and especially aripiprazole have well-documented antidepressant effects in the treatment of SCZ and depressive disorders [59,60,61]. However, depressive symptoms do not improve in all SCZ patients following antipsychotic treatment, possibly due to genetic variability [60]. For example, the 5-HT1A receptor gene functional polymorphism (rs6295) has been associated with the improvement of depressive symptoms in patients taking aripiprazole [62]. Several studies have demonstrated the effect of antipsychotic drugs on an increase in HSP90α expression [22,26,27]. This may indicate that an increase in HSP90 expression may have beneficial effects in patients with SCZ and other psychiatric disorders. Ishima et al. [22] found that aripiprazole, through the activation of 5-HT1A receptors, potentiated an NGF-induced neurite outgrowth in PC12 cells by increasing the HSP90α level. A recent study showed that a higher level of the HSP90 protein that is induced by antipsychotics (olanzapine, aripiprazole and blonanserin) contributes to the recovery of genesis of the GABAergic interneurons from NG2(+) cells. They also found that 17-AAG, an inhibitor of HSP90, attenuated the antipsychotic-induced GABAergic interneuron differentiation against the impairments that are caused by MK-801 [27]. GABAergic dysfunction is thought to be associated with the pathophysiology of SCZ [63,64] and major depressive disorder [65,66]. Moreover, the GABAergic neurons play an important role in the stress response through the regulation of the HPA axis [66]. Therefore, we speculate that the *HSP90AA1* rs11621560, rs4947 and rs2298877 polymorphisms may be associated with a poorer response to antipsychotic treatment and, as a result, higher PANSS emotional distress scores. In the case of the rs11621560 polymorphism, carriers of the least frequent C/C genotype had the highest score in the EMO factor. For the rs2298877 and rs4947 SNPs, we observed that the homo- and heterozygous carriers of the minor T and G alleles, respectively, had higher mean EMO factor scores than the major allele homozygotes. Unfortunately, we did not have data regarding the antipsychotic and antidepressant medication status of the patients (drug class, dose) that would allow us to perform additional statistical analyses to verify that the genotype-EMO associations that were observed did not reflect patterns of drug response. There is also no other study that explored the association of the *HSP90AA1* gene polymorphisms with the symptoms of SCZ or the response of SCZ patients to antipsychotic medications. Moreover, the functional significance of the polymorphisms in *HSP90AA1* has not yet been examined. Therefore, how rs11621560, rs4947 and rs2298877 polymorphisms influence the PANSS scale factors remains to be determined. To predict the possible impact of SNPs on gene structure and function, various in silico prediction programs (FuncPred, RegulomeDB, GTEx) that collect functional information using various tools and resources can be used [67]. The rs11621560 and rs2298877 polymorphisms are intronic variants, whereas rs4947 is a synonymous variant. SNPs in the intron regions may modulate mRNA splicing activity and influence the binding and function of long non-coding RNAs [68]. Synonymous variants, in turn, can disrupt transcription, splicing and mRNA stability and can also affect protein translation [69,70].

The following limitations should be considered when interpreting the results of the current study. First, owing to the relatively small sample size of the SCZ group, the frequencies of some homozygous genotypes were low in the subgroups after the stratified analyses, which may contribute to false-negative or false-positive results. Second, we focused on only the five SNPs of the *HSP90AA1* gene, which did not permit a complete coverage of this gene. Therefore, we cannot exclude that other polymorphisms of this gene may be responsible for its association with SCZ. Third, we did not investigate the biological effect of the *HSP90AA1* variants, which is necessary to explain the observed association between the rs11621560, rs4947 and rs2298877 polymorphisms and the EMO dimension of the PANSS. A fourth important limitation was the inability to perform multivariate analysis to account for any potential covariates; e.g., antipsychotic and antidepressant medication status that might have strengthened our conclusions regarding genotype–EMO associations. Therefore, although our study suggests an association between the *HSP90AA1* polymorphisms and the EMO dimension of the PANSS, this finding is based on simple comparative statistics and should be considered to be preliminary. On the other hand, our sample was well characterised and highly homogenous in terms of ethnicity (Polish Caucasians) with the control group from the same ethnic population, which served to reduce the potential effect of population stratification. Moreover, the patients were assessed by experienced clinicians and none of the patients was newly diagnosed in order to prevent any discrepancies in the diagnosis and clinical measures. We also broadly analysed the psychopathology of SCZ in association with the *HSP90AA1* polymorphisms as well as their impact on suicide attempts.

## 5. Conclusions

The current study showed no association between the five *HSP90AA1* polymorphisms and the risk of schizophrenia in either the single-marker or haplotype analyses. However, three SNPs (rs11621560, rs4947 and rs2298877) were associated with the emotional distress dimension of the PANSS. This is the first study to explore the association between *HSP90AA1* polymorphisms and SCZ. Larger studies of independent datasets are needed to validate these initial findings and to further evaluate the impact of the SNPs in *HSP90AA1* on the risk and psychopathology of SCZ. Furthermore, functional investigations that explore the impact of specific SNPs on *HSP90AA1* gene regulation should be the critical next steps.

## Figures and Tables

**Table 1:** Genotype distribution and allele frequencies for the five *HSP90AA1* polymorphisms in the SCZ patients (n = 409) and control group (n = 679).

SNP	Genotype/ Allele	Total	χ^2^	*p*	Females	χ^2^	*p*	Males	χ^2^	*p*
		SCZ/Control n(%)			SCZ/Control n(%)			SCZ/Control n(%)		
**rs8005905**	A/A	341(83.4)/556(81.9)	0.42	0.81	141(85.5)/261(81.6)	1.78	0.41	200(82.0)/295(82.2)	0.14	0.93
	A/T	66(16.1)/119(17.5)			24(14.5)/57(17.8)			42(17.2)/62(17.3)		
	T/T	2(0.5)/4(0.6)			0/2(0.60)			2(0.8)/2(0.6)		
	A	748(91.4)/1231(90.6)	0.30	0.58	306(92.7)/579(90.5)	1.12	0.30	442(90.6)/652(90.8)	0	0.97
	T	70(8.6)/127(9.4)			24(7.3)/61(9.5)			46(9.4)/66(9.2)		
**rs10873531**	A/A	323(79.0)/534(78.6)	0.06	0.97	135(81.8)/249(77.8)	1.12	0.57	188(77.0)/285(79.4)	0.55	0.76
	A/G	82(20.0)/139(20.5)			28(17.0)/67(20.9)			54(22.1)/72(20.1)		
	G/G	4(1.0)/6(0.9)			2(1.2)/4(1.2)			2(0.8)/2(0.6)		
	A	728(89.0)/1207(88.9)	0	0.99	298(90.3)/565(88.3)	0.71	0.40	430(88.1)/642(89.4)	0.37	0.54
	G	90(11.0)/151(11.1)			32(9.7)/75(11.7)			58(11.9)/76(10.6)		
**rs11621560**	A/A	150(36.7)/272(40.0)	1.93	0.38	66(40.0)/131(40.9)	3.54	0.17	84(34.4)/141(39.3)	1.83	0.40
	A/C	202(49.4)/306(45.1)			80(48.5)/134(41.9)			122(50.0)/172(47.9)		
	C/C	57(13.9)/101(14.9)			19(11.5)/55(17.2)			38(15.6)/46(12.8)		
	A	502(61.4)/850(62.6)	0.27	0.60	212(64.2)/396(61.8)	0.42	0.51	290(59.4)/454(63.2)	1.62	0.20
	C	316(38.6)/508(37.4)			118(35.8)/244(38.2)			198(40.6)/264(36.8)		
**rs4947**	A/A	268(65.5)/467(68.8)	1.55	0.46	114(69.1)/222(69.4)	0.40	0.82	154(63.1)/245(68.2)	4.08	0.13
	A/G	125(30.6)/192(28.3)			46(27.9)/85(26.6)			79(32.4)/107(29.8)		
	G/G	16(3.9)/20(2.9)			5(3.0)/13(4.1)			11(4.5)/7(1.9)		
	A	661(80.8)/1126(82.9)	1.41	0.24	274(83.0)/529(82.7)	0	0.96	387(79.3)/597(83.1)	2.61	0.11
	G	157(19.2)/232(17.1)			56(17.0)/111(17.3)			101(20.7)/121(16.9)		
**rs2298877**	C/C	268(65.5)/466 (68.6)	1.31	0.52	114(69.1)/222(69.4)	0.63	0.73	154(63.1)/244(68.0)	3.93	0.14
	C/T	125(30.6)/192(28.3)			46(27.9)/84(26.2)			79(32.4)/108(30.1)		
	T/T	16(3.9)/21(3.1)			5(3.0)/14(4.4)			11(4.5)/7(1.9)		
	C	661(80.8)/1124(82.8)	1.20	0.27	274(83.0)/528(82.5)	0.01	0.91	387(79.3)/596(83.0)	2.41	0.12
	T	157(19.2)/234(17.2)			56(17.0)/112(17.5)			101(20.7)/122(17.0)		

**Table 2:** The results of the haplotype analysis of the five *HSP90AA1* SNPs (rs8005905, rs10873531, rs11621560, rs4947, rs2298877).

Haplotype	Total			Females			Males		
	Frequency	OR (95% CI)	*p*	Frequency	OR (95% CI)	*p*	Frequency	OR (95% CI)	*p*
**A–A–A–A–C**	0.6162	1.00	---	0.6211	1.00	---	0.6127	1.00	---
**A–A–C–A–C**	0.1834	0.96 (0.76–1.21)	0.74	0.1799	0.84 (0.59–1.20)	0.33	0.1859	1.07 (0.78–1.47)	0.66
**T–G–C–G–T**	0.0880	0.89 (0.64–1.24)	0.49	0.0843	0.68 (0.40–1.16)	0.16	0.0910	1.08 (0.71–1.65)	0.71
**A–A–C–G–T**	0.0861	1.33 (0.98–1.81)	0.07	0.0855	1.20 (0.75–1.90)	0.44	0.0865	1.47 (0.97–2.22)	0.07
**A–G–C–A–C**	0.0186	1.35 (0.72–2.53)	0.35	0.0213	1.17 (0.49–2.82)	0.72	0.0165	1.67 (0.67–4.17)	0.27
**rare**	0.0077	2.40 (0.56–10.32)	0.24	0.0079	1.52 (0.33–7.03)	0.59	0.0075	4.13 (0.62–27.70)	0.14

OR—odds ratio; CI—confidence interval.

**Table 3:** Associations between the three SNPs of *HSP90AA1* and emotional distress factor (EMO) scores under different genetic models.

SNP	Model	Genotype		Total		Females		Males	
				EMO Scores *	*p*	EMO Scores *	*p*	EMO Scores *	*p*
rs11621560	Co-dominant	A/A		21.64 (0.41)	0.06	20.67 (0.49)	**<0.05**	22.40 (0.60)	0.57
		A/C		22.09 (0.34)		21.80 (0.52)		22.28 (0.45)	
		C/C		23.46 (0.69)		23.79 (1.18)		23.29 (0.87)	
	Dominant	A/A		21.64 (0.41)	0.14	20.67 (0.49)	**<0.05**	22.40 (0.60)	0.87
		A/C-C/C		22.39 (0.31)		22.18 (0.48)		22.52 (0.40)	
	Recessive	A/A-A/C		21.90 (0.26)	**<0.05**	21.29 (0.36)	**<0.05**	22.33 (0.36)	0.30
		C/C		23.46 (0.69)		23.79 (1.18)		23.29 (0.87)	
	Over-dominant	A/A-C/C		22.14 (0.35)	0.92	21.36 (0.48)	0.54	22.68 (0.49)	0.55
		A/C		22.09 (0.34)		21.80 (0.52)		22.28 (0.52)	
rs4947/rs2298877	Co-dominant	A/A	C/C	21.66 (0.30)	**<0.05**	21.13 (0.43)	0.15	22.05 (0.42)	0.19
		A/G	C/T	22.94 (0.44)		22.67 (0.68)		23.09 (0.57)	
		G/G	T/T	23.38 (1.25)		21.60 (0.68)		24.18 (1.76)	
	Dominant	A/A	C/C	21.66 (0.30)	**<0.01**	21.13 (0.42)	0.06	22.05 (0.42)	0.09
		A/G-G/G	C/T-T/T	22.99 (0.41)		22.57 (0.62)		23.22 (0.54)	
	Recessive	A/A-A/G	C/C-C/T	22.06 (0.25)	0.30	21.57 (0.36)	0.99	22.40 (0.34)	0.26
		G/G	T/T	23.38 (1.25)		21.60 (0.68)		24.18 (1.76)	
	Over-dominant	A/A-G/G	C/C-T/T	21.75 (0.29)	**<0.05**	21.15 (0.41)	0.05	22.19 (0.41)	0.20
		A/G	C/T	22.94 (0.44)		22.67 (0.68)		23.09 (0.57)	

* Data are expressed as the mean with standard error (SE); *p* values in bold are statistically significant. For each genetic model, the function gives a matrix with the sample size and percentages for each genotype, the *p*-value corresponding to the likelihood ratio test that was obtained from a comparison with the null model, and the Akaike information criterion (AIC) of each genetic model.

## Data Availability

The data that support the findings of this study are available from the corresponding author upon reasonable request.
